# History of a Case of Hydrocephalus Internus, in Which Copious Depletion by the Lancet, &c. Was Successfully Employed

**Published:** 1808-03

**Authors:** John Hamm

**Affiliations:** Dover, Delaware


### *History of a Case of Hydrocephalus Internus, in which copious Depletion by the Lancet, &c. was successfully employed.* By Dr. JOHN HAMM, *of Dover, Delaware.*

RODNEY FISHER, youngest son of John Fisher, Esq. of this town, aged about 7 years, was seized on the 1st of April, 1805, with unequivocal symptoms of a dropsy of the brain. About six weeks before he was attacked, he received a violent contusion, which I saw myself, on his forehead, by falling on the frozen ground, which cut him to the bone. This soon healed, and he continued in good health till the 1st of April. The symptoms were as follow: drowsiness, continual nausea and vomiting, high fever; partial delirium, obstinate costiveness, severe head-ache, chiefly in the fore part of the head; the head-ache alternated with the vomiting; grinding his teeth, picking the bed clothes, and starting often in his sleep; his pulse full and hard, evidently indicating an inflammatory diathesis of the arterial system.

When these symptoms had continued about 24 hours, the pupils of his eyes were evidently dilated, one more than the other; the head-ache became more intolerable, the fever more raging, the pulse frequent, the breathing hurried and quick, and the face flushed; the interrupted slumbers or perpetual restlessness were now succeeded with a lethargic torpor, eyes half closed, insensible to the light, the vomiting ceased, and his pulse now became tense and slow. These symptoms manifestly indicating an internal dropsy of the brain, I ordered a smart dose of calomel and jalap, which not operating, he was repeatedly glystered till plentiful evacuations from the bowels were produced; an emetic was administered to cleanse his stomach, which was irritable with vain efforts to vomit; afterwards a sudorific a sudorific medicine, venesection to ℥ viij. blister to the neck, calomel, two grains every two hours with the intention of producing salivation, with ung. merc. fort. externally, on the throat, thighs, and legs, till his mouth should be affected. The blood was very sizy.

April 2nd. Better after venesection, &c. Pulse rose and became full.

April 3rd. Blisters to his temples; complains much of his head; mercury, internally and externally, continued, and the enema frequently repeated.

April 4th. No better; symptoms run high; venesection to ℥ viij. blood as sizy as before; mercury continued, but has produced no evident effects upon his system. Evening of the 4th, pulse full and hard; venesection to ℥vj; blood still sizy; gave fol. sennæ, which operated well; and appears somewhat better.

April 5th. Worse this morning; the mercury has produced no effects upon his system; comatose all day, the pupils of his eyes dilated, pulse soft and quick, which seemed to threaten an approaching dissolution. Evening of the 5th, better; pulse more regular since the last venesection; complains still of his head; blister to his forehead, which operated thoroughly; mercury externally continued, and pulv. jalap. in repeated small doses, to increase evacuations from the bowels.

April 6th. Better this morning; the calomel has taken some effect upon his gums; the jalap produced two stools, very plentiful and green, and brought away one large worm; his eyes better and more natural, and the pupils somewhat contracted; the epispastic to his forehead, which drew well, seemed to have done good, and produced an alleviation of the head-ache; called for something to eat for the first time since he was taken; his eyes have become sensible to the light; mercury continued, with pulv. jalap. in the evening.

April 7th. Not so well as yesterday; coma and restlessness returned; pulse a little tense and irregular; complains of his head again; great irritability of his eyes to the light; no further sign of a salivation; his gums are affected; stools very fetid and green since the jalap administered last evening; prescribed another dose of calomel and jalap; operated well, and he was not so comatose afterwards.

April 8th. Better this morning; inclined to eat.

April 9th. Much better; stopped all medicines.

April 10th. Not so well this morning; feverish and somewhat somewhat delirious and comatose; pulse slow and very feeble; judged it proper to prescribe tinct. cort. Peruv. opt. comp. with wine and water in a very moderate quantity.

April 11th. Considerably better since the prescription yesterday, and experienced manifest benefit; life seemed to have been saved by the medicines used; and bids fair for a speedy recovery.

April 12th. Appetite good; senses perfect; pulse healthy and regular.

April 13th. Recovering strength hourly; eats hearty, sleeps sound, and goes about the room.

July, 1806. This boy has enjoyed good health ever since.

